# Comparison of administration of clopidogrel with aspirin versus aspirin alone in prevention of secondary stroke after transient ischemic attack

**DOI:** 10.1186/s40169-019-0223-z

**Published:** 2019-03-04

**Authors:** Mojtaba Khazaei, Fateme Ghasemian, Mehrdokht Mazdeh, Mohammad Taheri, Soudeh Ghafouri-Fard

**Affiliations:** 1 Department of Neurology, Hamadan University of Medical Sciences, Hamadan, Iran; 2 Neurophysiology Research Center, Hamadan University of Medical Sciences, Hamadan, Iran; 3 Department of Medical Genetics, Shahid Beheshti University of Medical Sciences, Tehran, Iran; 4 Urogenital Stem Cell Research Center, Shahid Beheshti University of Medical Sciences, Tehran, Iran

**Keywords:** Stroke, Clopidogrel, Aspirin

## Abstract

**Background:**

Patients with transient ischemic attack (TIA) or minor stroke are at risk of strokes. One of the precautionary measures to reduce risk of stroke in these patients is administration of antiplatelet drugs.

**Methods:**

The aim of this study was comparison of clopidogrel with aspirin versus aspirin alone in prevention of stroke following TIA or acute minor stroke. This clinical trial study was carried out in Farshchian Hospital, Hamadan, Iran in 2018. Fifty-four patients with TIA, isolated visual attacks, and minor stroke were randomly assigned to receive aspirin 80 mg/day or aspirin 80 mg/day and clopidogrel 75 mg/day for 3 months.

**Results:**

After this period, patients were examined for occurrence of myocardial stroke, hemorrhagic/ischemic stroke and drug complications. Ischemic stroke or TIA occurred in 5 (18.5%) patients in clopidogrel–aspirin group and 10 patients (37.1%) in aspirin group (P = 0.12).

**Conclusion:**

The study suggests a trend toward superiority of administration of clopidogrel with aspirin versus aspirin alone in protection against secondary stroke. Studies in larger cohorts of patients are needed to verify these results.

## Background

Stroke is an incapacitating disorder commonly seen in the emergency departments all over the world [1]. Transient ischemic attacks (TIAs) are considered as important predictor of successive stroke and demise [2]. The increased risk of subsequent stroke in the three-month period after TIA has been estimated to be 18.5% which necessitates appropriate interventions [3]. Several studies have compared the effects of administration of different antiplatelet regimens in patients with former TIA. For more than two decades, aspirin has been acknowledged as an effective antiplatelet for decreasing the risk of secondary stroke [4]. Moreover, combination of clopidogrel and aspirin has been administered in different clinical settings for prevention of myocardial infarction, stroke, or mortality from cardiovascular events [5, 6]. The same dual regimen has been shown to be superior to administration of aspirin alone in prevention of stroke after TIA or acute minor stroke in a Chinese population [7]. However, the effect of such regimen in prevention of secondary stroke has not been assessed in Iranian population. A previous study in Iranian patients has assessed platelet aggregation and frequency of main adverse cardiovascular events 1 month after the angioplasty in patients treated with aspirin and clopidogrel. Authors reported complete response to clopidogrel in 65% of patients, intermediate resistance in 22% and complete resistance in the remaining. Notably, no main adverse cardiovascular event was detected [8]. Based on the substantial role of genetic and acquired factors in the determination of response to clopidogrel [9], population-based studies are needed to assess the effects of administration of clopidogrel with aspirin in reduction of risk of secondary stoke. Consequently, we conducted the current clinical trial to compare the effects of clopidogrel with aspirin versus aspirin alone in prevention of secondary stroke after TIA in Iranian patients.

## Materials and methods

### Patients

The current clinical trial was conducted in Neurology Department, Farshchian Hospital, Hamadan during 2018 (January–August). Patients who met the following criteria entered the study: (1) Diagnosis of thrombotic TIA (including isolated visual changes), (2) Scores less than 15 based on the National Institutes of Health Stroke Scale (NIHSS) [10], (3) ability to cooperate with the research team. Patients were excluded from the trial if they had any of the subsequent criteria: intracranial hemorrhage, main non ischemic brain disorder, contraindication to aspirin or clopidogrel, cardiac arrhythmia, valvular heart disease, any condition which necessitate carotid intervention. Consent form was filled out by all the individuals in this study and the study was confirmed by the local Ethical committee of Hamadan University of Medical Sciences (IR.UMSHA.REC.1397.301).

### Study design

This was a randomized, double blind, parallel group study conducted in a University-affiliated hospital in Iran. A total of 54 patients diagnosed with TIA or acute minor stroke were randomized into two groups receiving either clopidogrel and aspirin or aspirin alone. Minor stroke was defined as the presence of NIHSS of 5 or less. No placebo was taken instead of clopidogrel in the aspirin alone group. The α and β values were set at 5% and 10% respectively.

The appropriate sample size was estimated from the following equation:

$${\text{n}} = \frac{{Z_{{1 - \frac{\alpha }{2}}} \sqrt {\overline{P} \left( {1 - \overline{\text{P}} } \right)} + Z_{1 - \beta } \sqrt {P_{0} (1 - P_{0)} + P_{1} \left( {1 - P_{1} } \right)}^{2} }}{{\left( {P_{0} - P_{1} } \right)^{2} }} \cong 27$$

Z_1−β_ = 1.28, Z1_−α_ = 1.96, P_1_ = 10%, P_0_ = 18.5%

Risk of secondary stroke was considered as 18.5% for aspirin treated patients based on a previous study [3] and 10% for dual regimen [11].

Patients received either aspirin 80 mg/day or aspirin 80 mg/day and clopidogrel 75 mg/day for 3 months.

### Outcomes measured

The odds and risk ratio of secondary TIA was measured in a 3-month period as the main outcomes. The adverse effects of each regimen were also recorded.

### Statistical methods

Data were analyzed using SPSS v. 16 (IBM Corp, Chicago, IL, USA). P values less than 0.05 were considered as significant. Quantitative variables were described using mean and standard deviation (SD), whereas categorical variables were expressed as ratios or percentages. Chi square and student t tests were used for assessment of categorical and quantitative variables respectively.

## Results

There were no substantial differences in the baseline features of the two groups, including type of TIA, TIA severity and sex ratio (Tables 1 and 2). No significant difference was detected in age of study participants (68.9 ± 10.5 in aspirin group, 65.7 ± 13.5 in aspirin + clopidogrel group, P = 0.321, *df* = 52, t = 1). Moreover, there were no significant differences in basic patient characteristics such as diabetes, hypertension, smoking, renal insufficiency between two groups of patients (P values > 0.05).

**Table 1:** Sex ratio of study participants in each group

	Aspirin group	Aspirin + Clopidogrel group	Statistics
Male	20 (74.1%)	14 (51.8%)	P = 0.09 *df* = 1 χ^2^ = 2.85
Female	7 (25.9%)	13 (25.9%)

**Table 2:** Comparison of TIA type in study groups

	Aspirin group	Aspirin + Clopidogrel group	Statistics
TIA	20 (74.1%)	16 (59.3%)	P = 0.248 *df* = 1 χ^2^ = 1.3
Minor and isolated visual attacks	7 (25.9%)	11 (40.7%)	
Total	27 (100%)	27 (100%)	

There was no significant difference in occurrence of secondary TIA in 3 month period between two groups [37.1% in aspirin group, 18.5% in aspirin + clopidogrel group, P = 0.129, *df* = 1, χ^2^ = 2.3, OR (95% CI) = 2.6 (0.8–9), RR (95% CI) = 1.7 (0.8–3.6)]. Odds ratio and risk ratio of secondary TIA in each group are shown in Table 3.

**Table 3:** Odds ratio (OR) and risk ratio (RR) of secondary TIA in each group

Drugs	Occurrence of secondary TIA		
	Number (%)	OR (95% CI)	RR (95% CI)
Aspirin	10 (37.1%)	2.6 (0.8–9)	1.7 (0.8–3.6)
Aspirin + Clopidogrel	5 (18.5%)	1	1

Two patients in aspirin group and no patient in the second group subsequently had myocardial infarction (P = 0.150, *df* = 1, χ^2^ = 2.07). Hemorrhagic stroke occurred in one patient in aspirin + clopidogrel group but no patient in the other group. There was no significant difference in adverse effects of drugs between two groups. All occurred adverse events are listed in Table 4.

**Table 4:** The frequencies of adverse drug effects in each group

	Aspirin group	Aspirin + Clopidogrel group	P value
Gastrointestinal hemorrhage	1 (3.7%)	2 (7.4%)	NS
Other gastrointestinal complications^a^	4 (14.8%)	1 (3.7%)	NS
Hemorrhagic stroke	0 (0%)	1 (3.7%)	NS
Other hemorrhagic events	0 (0%)	0 (0%)	NS

*NS* not significant

^a^Heartburn, dyspepsia, epigastric discomfort, bloating, and early satiety

## Discussion

In spite of recent advances in diagnosis and treatment of cerebral ischemic events, these conditions are associated with both mortality and functional dependence [12]. Consequently, several studies have focused on identification of preventive modalities for their occurrence. Most of patients with ischemic stroke experience further episodes of stroke or TIA, instead of myocardial infarction. Consequently, antiplatelet therapies are suggested for prevention of secondary stroke [13]. The US Food and Drug Administration (FDA) have approved 4 drugs for protection against secondary stroke: monotherapy with aspirin, ticlopidine, clopidogrel, and dual administration of aspirin and extended-release dipyridamole [14]. Combinatory regimens with the aim of highest platelet inhibition in addition to vascular protection are proposed as the best strategy in this regard [13].

In the present study, we compared effects of clopidogrel with aspirin versus aspirin alone in the prevention of secondary stroke after TIA in a group of Iranian patients. Although patients received the combinatory regimen had lower occurrence of secondary stroke, the difference between two groups did not reach the significance level. Previous studies have reported superiority of combinatory regimen over aspirin alone in this regard in other populations [7]. Lack of significant difference between two groups in our current study might be explained by low sample size of the study. Alternatively, genetic- or ethnic-based differences in response to clopidogrel might be involved in the observed lower efficacy of this drug in Iranian patients.

Based on the acknowledged effects of CYP2C19 genetic polymorphism in determination of response to clopidogrel [15], FDA has recommended that clopidogrel must be administered with sufficient precautions in populations with a high prevalence of risk alleles [16]. Clopidogrel is activated in two phases and CYP2C19 has a critical role in this process. Genetic polymorphisms in the coding gene may result in the emergence of loss-of-function alleles, CYP2C19*2 and CYP2C19*3, and successively to the lack of the enzyme activity [17]. A previous study in Iranian population have shown relative high frequency of risk alleles in Iranian patients and stated that the FDA recommendations are more beneficial to be adapted in this country compared with other regions [18]. The frequencies of CYP2C19*2 and *3 were 23.4% and 3.9% in Iranian people, respectively [18]. However, the frequencies of CYP2C19 *2 and *3 have been 0.11–0.16 and 0.0–0.7 in Caucasian [19], 0.11% and 0.002% in Egyptians, 0.15% and 0.01% in Israeli Jews [20], and 0.13% and 0.03% in Lebanese [21], respectively. Another study has shown complete resistance in 13% of Iranian patients treated with clopidogrel which was not significantly different from other countries [8]. Future multi-center studies with larger sample sizes are necessary to explore the efficacy of mentioned combinatory regimen in prevention of secondary stroke in Iranian patients.

## Conclusion

Based on our results, the frequency of hemorrhagic stroke and myocardial stroke as secondary events following TIA was not different between the study groups. Moreover, the combinatory regimen was not associated with higher rate of adverse effects. Consequently, our study demonstrates a trend toward superiority of administration of clopidogrel with aspirin versus aspirin alone in protection against secondary stroke after TIA in Iranian population and warrants future studies with larger sample sizes.
